# Congenitally Corrected Transposition of the Great Arteries in Adults—A Contemporary Single Center Experience

**DOI:** 10.3390/jcdd8090113

**Published:** 2021-09-15

**Authors:** Josef Auer, Claudia Pujol, Susanne J. Maurer, Nicole Nagdyman, Peter Ewert, Oktay Tutarel

**Affiliations:** 1 Department of Congenital Heart Disease and Paediatric Cardiology, German Heart Centre Munich, TUM School of Medicine, Technical University of Munich, 80636 Munich, Germany; auer@dhm.mhn.de (J.A.); claupujol@gmail.com (C.P.); nagdyman@dhm.mhn.de (N.N.); ewert@dhm.mhn.de (P.E.); 2 Department of Electrophysiology, German Heart Centre Munich, TUM School of Medicine, Technical University of Munich, 80636 Munich, Germany; susanne.maurer@tum.de; 3 DZHK (German Centre for Cardiovascular Research), Partner Site Munich Heart Alliance, 80992 Munich, Germany

**Keywords:** adult congenital heart disease, transposition of the great arteries, mortality

## Abstract

Background: Congenitally corrected transposition of the great arteries (ccTGA) is a rare congenital heart defect (CHD). Contemporary data regarding its outcome in adults are scarce. Methods: Retrospective, single-center study of all ccTGA patients over the age of 16 years treated at our center during the time period 2006–2018. Only patients with a biventricular circulation were included. The primary endpoint was all-cause mortality. Results: Altogether, 96 patients (mean age 32.8 ± 16.0 years, female 50%) with ccTGA and a systemic right ventricle (SRV) were included in the study. An additional CHD was present in 81 patients (84.4%); most common were a ventricular septal defect (VSD) and a left ventricular outflow tract obstruction. Out of the whole cohort, 45 (46.9%) had already undergone cardiac surgery at baseline. During a median follow-up of 6.5 (IQR 2.8–12.7) years, the primary endpoint occurred in 10 patients (10.8%). Cause of death was cardiac in nine patients and suicide in one. Hospitalizations due to heart failure occurred in 48 patients (51.6%). Upon univariate Cox analysis, an NYHA class ≥III, severe tricuspid regurgitation, severe SRV systolic impairment, as well as a reduced left ventricular systolic function were predictors of the primary endpoint. Upon multivariable analysis, only NYHA class ≥ III (HR: 18.66, CI 95%: 3.01–115.80, *p* = 0.0017) and a reduced left ventricular systolic function (HR: 7.36, CI 95%: 1.18–45.99, *p* = 0.038) remained as independent predictors. Conclusions: Adults with ccTGA and an SRV are burdened with significant morbidity and mortality. Predictors for mortality are NYHA class and subpulmonary left ventricular function.

## 1. Introduction

Congenitally corrected transposition of the great arteries (ccTGA) is characterized by discordant atrio-ventricular and ventriculo-arterial connections [1]. Thus, the morphologically right ventricle supports the systemic circulation and is described as a systemic right ventricle (SRV), while the morphologically left ventricle is the subpulmonary ventricle. It is a rare congenital heart defect (CHD), with an estimated prevalence of 1 per 33,000 live births, thus accounting for approximately only 0.05% of all CHD [2]. 

Data regarding the clinical course of ccTGA patients are scarce, especially in adult patients [3]. One of the largest studies reported so far was an international multicenter cross-sectional study that included 182 patients from 19 centers [4]. It reported an increasing incidence of SRV dysfunction and clinical congestive heart failure, especially in those patients with associated lesions, such as a large ventricular septal defect [4]. However, this study lacked follow-up data. Furthermore, it was published more than 20 years ago, probably reflecting patients from a different era preceding current developments regarding medical, interventional and surgical therapies [5]. Data from a contemporary cohort are lacking. Therefore, the aim of this study was to describe a contemporary cohort of adult ccTGA patients and to evaluate predictors of outcome.

## 2. Materials and Methods

This retrospective, single-center study included all patients with a ccTGA diagnosis under follow-up at the German Heart Centre Munich who were ≥16 years of age and were treated at our center during the time period 2006 until 2018. Only patients with a biventricular circulation were included, excluding for example patients with a single-ventricle physiology. The time point of inclusion and the commencement of follow-up was the first clinical appointment during the study period, if the patient was already 16 years of age or older, or the first clinical appointment after the 16th birthday, if the patient reached this age-threshold during the study period. 

Information regarding medical/surgical history as well as demographic data were retrieved from hospital records. All catheter interventions as well as surgeries including device implantations at baseline and during follow-up were recorded. Symptomatic status was assessed according to the New York Heart Association classification (NYHA). Arrhythmias included any type of supraventricular and ventricular arrhythmias requiring treatment. Cyanosis was defined as a resting oxygen saturation below 90%. Heart failure was diagnosed according to recent guidelines [6]. 

Echocardiography was performed by experienced operators. A semi-quantitative assessment of left and right ventricular systolic function via multiview 2-dimensional echocardiography was used, grading it as normal, or mildly, moderately or severely impaired, as previously described [7]. More advanced methods such as strain analysis could not be applied due to the retrospective nature of the data. Tricuspid regurgitation was assessed according to recent guidelines [8,9]. The primary endpoint was all-cause mortality. 

Statistical analyses were performed using SPSS version 25 (IBM Corp., Armonk, NY, USA) and MedCalc version 20 (MedCalc Software, Ostend, Belgium). Continuous variables are presented as mean ± standard deviation or median (interquartile range), whereas categorical variables are presented as number (percentage). Comparison between groups was performed using the Mann–Whitney U test or Student’s *t*-test for continuous, and the Chi-square test or Kruskal–Wallis test for categorical variables. Univariate Cox proportional hazards analysis was used to assess the association between variables and the primary endpoint. Significant variables (*p* < 0.05) from this analysis were included in a multivariate Cox proportional hazards analysis model in a stepwise fashion.

Kaplan–Meier curves and log-rank test were used to compare event-free survival from the primary endpoint for the significant variables from the multivariate Cox analysis. All tests were performed two-sided. For all analyses a *p*-value < 0.05 was considered statistically significant. The Sankey diagrams were drawn using the Sankey diagram builder at sankeymatic.com (accessed on 26 July 2021).

## 3. Results

Altogether, 99 patients with ccTGA were included in the study. Out of these, three patients had an anatomic correction. Due to the small number of patients with an anatomic correction, these were excluded from the further analysis. The remaining 96 patients (mean age 32.8 ± 16.0 years, female 50%) all had an SRV. An additional CHD was present in 81 patients (84.4%); most common were a VSD and a left ventricular outflow tract obstruction (LVOTO). Out of the whole cohort, 45 (46.9%) had already undergone cardiac surgery at baseline. These included physiological corrections with VSD closures, while five patients had already received a tricuspid valve prosthesis. The majority of patients reported a good subjective exercise capacity at baseline, with around 80% in NYHA class I and II. More detailed information regarding baseline characteristics is presented in Table 1 and Table 2.

### 3.1. Follow-Up

**Figure 1:**
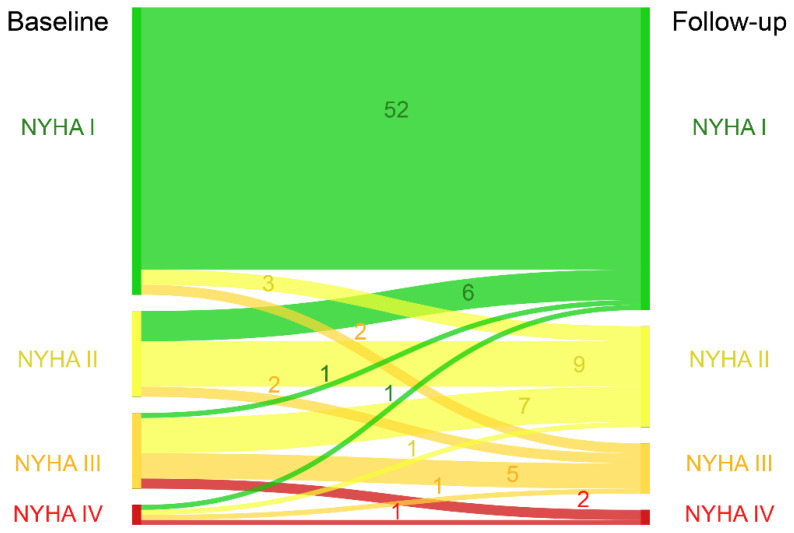
Sankey diagram of New York Heart Association class at baseline and follow-up.

A follow-up was available in 93 patients (96.9%). After a median follow-up of 6.5 (IQR 2.8–12.7) years, 86% of patients were in NYHA class I or II (Figure 1). 

**Figure 2:**
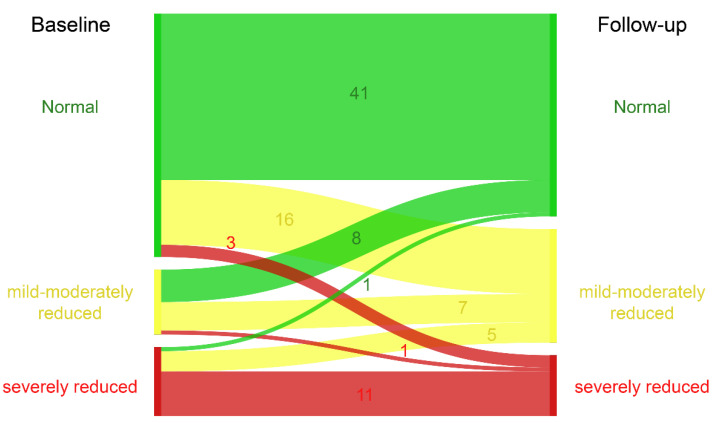
Sankey diagram of systolic systemic ventricular function at baseline and follow-up.

While 34% had a reduced SRV function at baseline, this number increased to 46% at the last follow-up (Figure 2). 

**Figure 3:**
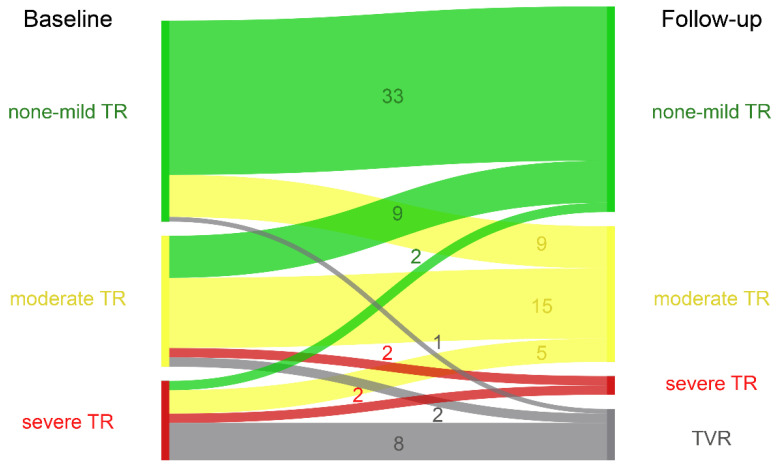
Sankey diagram of tricuspid regurgitation at baseline and follow-up.

During follow-up, 19 patients underwent cardiac surgery, which in the majority of cases was tricuspid valve surgery (11 replacements, four reconstructions; Figure 3). 

New atrial arrhythmias occurred in 11 patients (11.8%), while ventricular tachycardias were present in 9 (9.7%). A new pacemaker was implanted in 18 patients (19.4%), while complete heart block developed in 6 (6.5%). Catheter interventions were necessary in 12 patients (12.9 %) and electrophysiological ablations in 13 (14.0%). Hospitalizations due to heart failure occurred in 48 patients (51.6%). Three patients received a heart transplantation; two out of these died during the postoperative course after the transplantation. The primary endpoint occurred in 10 patients (10.8%). Cause of death was cardiac in nine patients and suicide in one.

### 3.2. Predictors of Primary Endpoint

**Figure 4:**
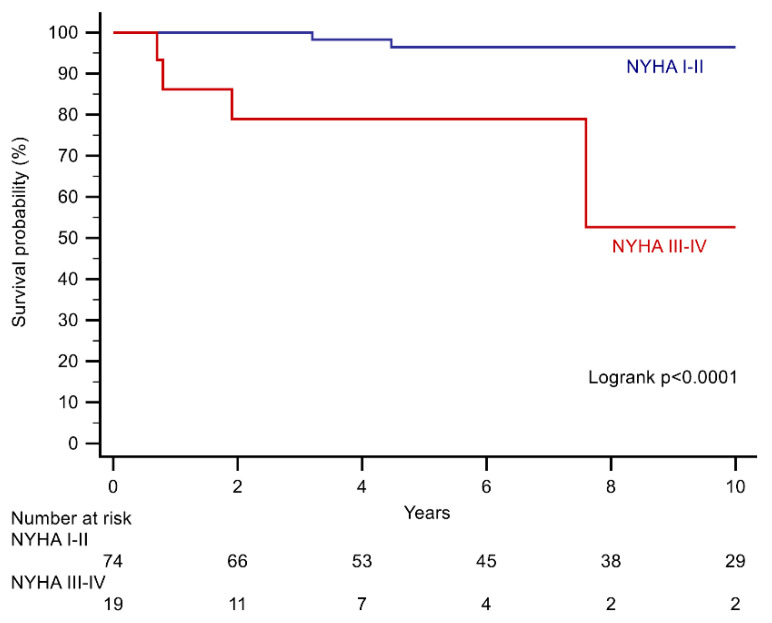
Kaplan–Meier curves stratifying patients according to NYHA class at baseline.

**Figure 5:**
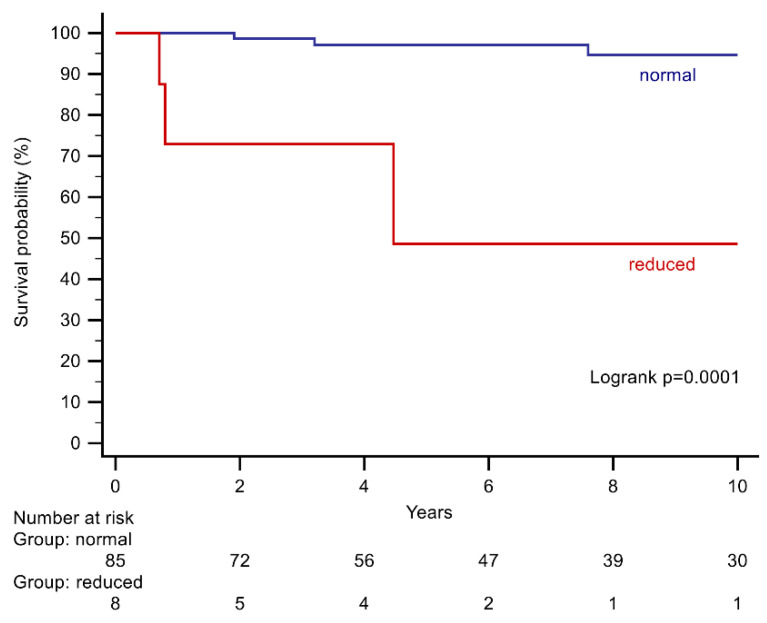
Kaplan–Meier curves stratifying patients according to systolic left ventricular function at baseline.

Upon univariate Cox analysis, an NYHA class ≥III, severe tricuspid regurgitation, severe SRV systolic impairment, as well as a reduced left ventricular systolic function were predictors of the primary endpoint (Table 3). Upon multivariable analysis, only NYHA class ≥ III (HR: 18.66, CI 95%: 3.01–115.80, *p* = 0.0017), and a reduced left ventricular systolic function (HR: 7.36, CI 95%: 1.18–45.99, *p* = 0.038), remained as independent predictors (Table 3, Figure 4 and Figure 5).

## 4. Discussion

In this retrospective single-center study, adult ccTGA patients were burdened with significant morbidity and mortality. Subjective exercise limitations as well as a reduced systolic function of the subpulmonary left ventricle predicted mortality.

Generally, a good subjective exercise capacity was reported by the patients in this study, with 80% being in NYHA class I and II. However, in those with NYHA class III or IV, the risk of mortality was more than 18-fold higher (Table 3). In a large study from the Royal Brompton Hospital in London/UK, Bredy and colleagues recently reported NYHA class as an important prognostic marker in ACHD patients [10]. Altogether, 2781 ACHD patients were studied, including 216 patients with an SRV, and NYHA class emerged as a strong predictor of mortality, with an 8.7-fold increased mortality risk in class III patients compared with class I patients [10]. Additionally, functional class was also a predictor of a worse clinical outcome in a large cohort of ACHD patients over the age of 60 years [11]. Our study confirms these results and emphasizes the importance of NYHA class in the clinical assessment of ccTGA patients. Interestingly, there was a strong relation between NYHA class and objective measures of exercise capacity derived from cardiopulmonary exercise testing in the study by Bredy and colleagues [10]. Still, 55% of patients in NYHA class I had a percent predicted peak VO_2_ below 80%, and were thus impaired, despite being asymptomatic [10]. This points to the limitations of the NYHA classification, especially in asymptomatic or mildly symptomatic ACHD patients. However, if a patient is limited to the point that an NYHA class of III or more is reported, this is an alarming sign. Unfortunately, cardiopulmonary exercise testing data at baseline were not available in a large proportion of our patients, and could therefore not be included into the evaluation.

While a reduced SRV function and severe TR are well described in ccTGA patients, recently, attention has shifted to the subpulmonary left ventricle [12]. In a retrospective analysis, transthoracic echocardiography studies of 157 patients with an SRV (43.3% with a ccTGA) were studied [12]. The majority of patients (84.7%) were in NYHA class I or II, as in our study, and 86.6% had a normal systolic function of the subpulmonary LV [12]. This was again similar to our study (91.7%). Interestingly, upon multivariable logistic regression analysis, LV systolic dysfunction was most strongly associated with NYHA class III or IV [12]. This, together with our finding that a reduced systolic LV function is a predictor of mortality, shines a light on the importance of the LV in patients with an SRV. Until now, there has not been much emphasis on the subpulmonary LV in these patients [12], but in the light of these findings, this probably needs to change to facilitate a better risk assessment in ccTGA patients. The reasons for LV impairment in this setting are multifactorial. The morphologically LV in ccTGA patients is a fragile ventricle sensitive to hemodynamic changes in pre- and afterload, and especially dysfunction of the SRV [12]. Post-capillary pulmonary hypertension due to longstanding SRV dysfunction might also play a role, as well as in some cases volume overload from mitral regurgitation [13]. Additionally, intrinsic myocardial disease is probably also an important contributor [13]. A question that needs to be answered is whether LV systolic dysfunction is just a late manifestation of heart failure in patients with SRV, or is an independent marker preceding and predicting a worsening of symptoms [14]. At least in our study, it was an independent predictor of mortality.

As pointed out, a reduced SRV function and severe TR are well-known complications in ccTGA patients. Around 55–70% of patients have SRV dysfunction with a continuing upsurge as patients are getting older, and 40–57% have moderate to severe TR [4]. In keeping with these reported numbers, 34% of patients in our study had a reduced SRV function at baseline, and this number increased to 46% at the last follow-up. SRV dysfunction has severe clinical implications. SRV failure was described as the cause of death in >50% of the patients who died in one series, and was accompanied by severe TR [3]. Both tricuspid valve abnormalities and the dilatation of the SRV contribute to the development of TR. In this scenario, severe TR is more than an innocent bystander and should be treated accordingly. Tricuspid valve replacement is recommended in symptomatic patients with severe TR and preserved or mildly impaired systemic RV systolic function (EF > 40%), while it should be considered in asymptomatic patients with severe TR and progressive SRV dilatation and/or mildly impaired SRV systolic function (EF > 40%) [15]. In patients with more severe SRV dysfunction, which bears a prohibitive surgical risk, percutaneous interventional techniques such as edge-to-edge repair might offer an option [5].

During a 6.5-year follow-up, a new pacemaker was necessary in 19.4% of patients, equaling around 3% per year. Complete heart block develops in ccTGA patients mainly due to the abnormal disposition of the atrioventricular conduction system [16]. Its incidence increases as the patients get older, and is estimated to be between 30 and 38% in older children and adults with ccTGA [16]. The yearly rate of new-onset complete heart block has been reported as approximately 2% per year [17]. Therefore, the requirement for pacemaker implantation will increase as patients are getting older, as also confirmed in our study. However, a caveat is that late-onset systemic ventricular dysfunction can occur with the use of univentricular pacing, and therefore, primary biventricular pacing might be a better option in these patients [18].

A limitation of our study is its retrospective design, which has some obvious limitations. For example, data regarding the occurrence of complete heart block perioperatively during VSD closure were incomplete. Additionally, variables such as data from exercise testing and laboratory analyses (e.g., brain natriuretic peptides) were not available in all patients, and could therefore not be included into the analysis. Furthermore, data on patients with anatomic repair were limited. With the more widespread adoption of these surgical techniques, the face of adult ccTGA might change, but this is not without its own challenges [1]. However, we contend that our study presents the largest single-center cohort with contemporary data currently available.

## 5. Conclusions

Adults with ccTGA and an SRV are still burdened with significant morbidity and mortality. Better medical, interventional and surgical treatment options need to be developed for this emerging population.

## Figures and Tables

**Table 1:** Baseline characteristics.

	All	Alive	Dead	*p*
n	96	86	10	
Age (years)	32.8 ± 16.0	32.2 ± 15.9	37.9 ± 16.8	0.325
Female, n (%)	48 (50.0)	44 (51.2)	4 (40.0)	0.504
Situs Inversus	11 (11.5)	8 (9.3)	3 (30.0)	0.052
Additional Lesions (all)	81 (84.4)	72 (83.7)	9 (90.0)	0.605
VSD	56 (58.3)	48 (55.8)	8 (80.0)	0.142
LVOTO	55 (57.3)	51 (59.3)	4 (40.0)	0.243
Ebstein	13 (13.5)	12 (14.0)	1 (10.0)	0.729
Extracardiac Comorbidity	59 (61.5)	53 (61.6)	6 (60.0)	0.920
Previous Cardiac Surgery	45 (46.9)	40 (46.5)	5 (50.0)	0.834
History of Atrial Arrhythmias	29 (30.2)	24 (27.9)	5 (50.0)	0.150
History of Ventricular Arrhythmias	8 (8.3)	8 (9.3)	0 (0)	0.314
History of Electrophysiological Ablation	5 (5.2)	4 (4.7)	1 (10.0)	0.471
NYHA class, n (%)				**0.042**
I	59 (61.5)	55 (64.0)	4 (40.0)	
II	18 (18.8)	17 (19.8)	1 (10.0)	
III	15 (15.6)	12 (14.0)	3 (30.0)	
IV	4 (4.2)	2 (2.3)	2 (20.0)	
Pacemaker	25 (26.0)	20 (23.3)	5 (50.0)	0.068
ICD	2 (2.1)	2 (2.3)	0 (0)	0.626

NYHA: New York Heart Association; VSD: ventricular septal defect; LVOTO: left ventricular outflow tract obstruction.

**Table 2:** Echocardiography at baseline.

	All	Alive	Dead	*p*
Systemic Right Ventricular Systolic Function				**0.028**
Normal	63 (65.6)	59 (68.6)	4 (40.0)	
Mild–Moderately Reduced	16 (16.7)	15 (17.4)	1 (10.0)	
Severely Reduced	17 (17.7)	12 (14.0)	5 (50.0)	
Left Ventricular Systolic Function				**0.011**
Normal	88 (91.7)	81 (94.2)	7 (70.0)	
Mild–Moderately Reduced	4 (4.2)	2 (2.3)	2 (20.0)	
Severely Reduced	4 (4.2)	3 (3.5)	1 (10.0)	
Tricuspid Regurgitation				**0.021**
None–Mild	44 (45.8)	42 (48.8)	2 (20.0)	
Moderate	30 (31.3)	29 (33.7)	1 (10.0)	
Severe	17 (17.7)	12 (14.0)	5 (50.0)	
Tricuspid Valve Prosthesis	5 (5.2)	3 (3.5)	2 (20.0)	0.220

**Table 3:** Univariate and multivariate analysis of predictors for all-cause mortality.

	Univariate		Multivariate	
Variable	HR (95% CI)	*p*	HR (95% CI)	*p*
Age	1.04 (0.9998–1.09)	0.051		
Additional Lesions	0.99 (0.12–8.10)	0.995		
VSD	2.93 (0.62–13.85)	0.174		
Male	1.37 (0.39–4.87)	0.623		
Pacemaker	2.82 (0.81–9.77)	0.103		
**NYHA class ≥ III**	**15.06 (3.43–66.03)**	**<0.001**	**18.66 (3.01–115.80)**	**0.0017**
Severe TR	16.78 (3.19–88.22)	<0.001		
Severe SRV Dysfunction	14.48 (3.29–63.67)	<0.001		
**Reduced LV Function**	**10.84 (2.52–46.67)**	**0.001**	**7.36 (1.18–45.99)**	**0.038**

HR: hazard ratio; NYHA: New York Heart Association; VSD: ventricular septal defect; TR: tricuspid valve regurgitation; SRV: systemic right ventricle; LV: left ventricle.

## Data Availability

The data associated with this paper are not publicly available.
